# Early motor behaviour in infants born to mothers in opioid replacement therapy during pregnancy: a prospective cohort study

**DOI:** 10.1186/s12887-026-07416-y

**Published:** 2026-07-31

**Authors:** Bodil B. Singstad, Merete Brandal, Vibeke S. Aulie, Tordis Ustad, Lars Adde, Toril Fjørtoft, Kari Anne I. Evensen

**Affiliations:** 1 Clinic of Rehabilitation, St. Olavs hospital, Trondheim University Hospital, Trondheim, Norway; 2 Department of Rehabilitation Science and Health Technology, Oslo Metropolitan University, Oslo, Norway; 3 Division of Paediatric and Adolescent Medicine, Oslo University Hospital, Oslo, Norway; 4 Department of Clinical and Molecular Medicine, Norwegian University of Science and Technology, Trondheim, Norway; 5 Children`s Clinic, St. Olavs Hospital, Trondheim University Hospital, Trondheim, Norway

**Keywords:** Prenatal exposure, Opioid replacement therapy, Early motor behaviour, General movement assessment, Motor optimality score revised

## Abstract

**Background:**

There is a strong recommendation for opioid replacement therapy (ORT) in pregnant women dependent on opioids. Using drugs and alcohol in pregnancy is a well-documented risk factor for later neurodevelopmental impairment, and the infants have reduced quality of their early motor behaviour at the age of 3–4 months corrected age (CA). Growing evidence indicates that ORT exposure in utero has negative long-term effects on the child but whether ORT-exposed infants have reduced quality of early motor behaviour has never been studied. The primary aim of this study was to examine early motor behaviour at 3–4 months CA in infants born to mothers in ORT during pregnancy, infants exposed to alcohol and/or drugs and unexposed infants. Secondary, we aimed to estimate odds for reduced motor behaviour in the three groups.

**Methods:**

In this prospective cohort study, Children Exposed to Alcohol and/or Drugs in Intrauterine Life (CEADIL), we used cross-sectional data to compare a group of infants exposed to ORT during pregnancy (ORT group, *n* = 15), a group of infants exposed to alcohol and/or drugs during pregnancy (CEADIL group, *n* = 99) and a group of healthy unexposed infants (control group, *n* = 105). Outcome measures were Motor Optimality Score-Revised (MOS-R) and the Alberta Infant Motor Scale (AIMS) at 3–4 months CA.

**Results:**

The median MOS-R score was 26 (IQR 22-28) in the ORT group and 26 (IQR 24–28) in the CEADIL group, compared with 28 (IQR 26-28) in the unexposed control group (*p* < 0.001). The ORT and the CEADIL group had higher odds for a MOS-R total score ≤ 24, a higher frequency of aberrant fidgety movements, reduced or absent age-adequate movement repertoire, abnormal observed postural patterns, and abnormal movement character, compared with the unexposed control group. The AIMS total score was 8.0 (IQR 7.0-11.5) in the ORT group and 9.0 (IQR 8.0-10.0) in the CEADIL group, compared with 11.8 (IQR 11.0-13.0) in the unexposed control group (*p* < 0.001).

**Conclusions:**

Similar to the CEADIL group, the ORT group had reduced quality of early motor behaviour compared with the control group. Hence, ORT-exposed infants may be at risk for neurodevelopmental impairment.

**Trial registration:**

Not applicable.

## Background

There is a strong recommendation for opioid replacement therapy (ORT) in pregnant women dependent on opioids, both in national [1] and international guidelines [2–4]. ORT has been used since the 1950s, and the medical therapy consists of a daily dose of methadone or buprenorphine as a liquid or pill [5]. The treatment is multidisciplinary and the goal of ORT is to achieve the best possible health, functional and coping skills, and quality of life [6]. ORT during pregnancy reduce maternal illicit opioid use, improve prenatal care and reduce maternal symptoms of craving and withdrawal [7].

Infants born to mothers in ORT during pregnancy have lower birth weight and birth length, and have smaller head circumference compared with unexposed children at birth [1]. The Norwegian guidelines indicate that it is unclear whether the causality is because of the medical treatment (ORT) or secondary to the mother’s lifestyle. Neonatal Abstinence Syndrome (NAS) is detected in 60–90% of the ORT-exposed children after birth, and half of these need medical treatment [1]. The symptoms of NAS decrease during the child’s first months of life, but disturbances in the central nervous system can persist until 4–6 months of age [8].

Use of drugs and alcohol in pregnancy is a well-documented risk factor for later neurodevelopmental impairment (NDI) in the offspring [9–15]. However, there is a lack of conclusive knowledge on long-term consequences for neurodevelopment in children born to mothers in ORT, although growing evidence indicate negative long-term effects for the child [9, 13, 16–18]. Reported long-term consequences described in these meta-analyses and studies are visual problems, adverse psychomotor and motor functioning, more behavioural problems, impaired attention and executive functioning compared with unexposed children.

Early identification of infants at risk for NDI can facilitate targeted health services from an early age, and there is growing evidence that early, targeted intervention can improve functional outcomes for these infants [19]. The General Movement Assessment (GMA) is a cost-effective and highly reliable tool for detecting abnormal neurological development in young high-risk infants [20]. An increasing number of studies have found that GMA in high-risk-children can also predict later cognitive dysfunction in children not developing cerebral palsy (CP) [15, 21, 22]. Fidgety Movements (FMs) cannot, as far as we know, predict cognitive function, but subcategories of concurrent movements and posture in combination with FMs can [21]. Motor Optimality Score-Revised (MOS-R) is the sum score of these subcategories [23]. Emerging evidence suggests that the MOS-R can be used as a screening tool due to its potential to identify children at risk for adverse neurological outcomes, even when they exhibit normal fidgety movements [24–26].

In a previous study, Children Exposed to Alcohol and/or Drugs in Intrauterine Life (CEADIL), we have shown that exposed infants have an abnormal movement character and higher odds for atypical motor development [14]. At follow-up at two years, we found that the exposed children had poorer neurodevelopmental outcomes, compared with an unexposed, healthy control group [15]. Another study has shown that prenatal exposure to opioid and Human Immunodeficiency Virus is associated with abnormal early spontaneous movement by abnormal general movements [27]. However, to our knowledge, whether ORT-exposed infants have abnormal motor behaviour has never been studied.

The primary aim of this study was to examine early motor behaviour at 3–4 months corrected age (CA) in infants born to mothers in ORT during pregnancy (ORT group), infants exposed to alcohol and/or drugs (CEADIL group) and a group of unexposed infants (control group). The secondary aim was to estimate odds for reduced motor behaviour in the three groups.

## Methods

### Study design

The present study is part of the CEADIL study analysing cross-sectional data on infants at 3–4 months CA. The CEADIL study is a prospective cohort study within the follow-up program for infants at risk for impaired development after prenatal exposure of ORT, alcohol and/or drugs at St. Olavs hospital, Trondheim University Hospital and Oslo University Hospital, Ullevål, both in Norway. The participants in the ORT group and the CEADIL group were enrolled consecutively between 6 December 2014 and 5 May 2019. To ensure an adequate sample size, the inclusion period for the ORT group was further extended until 31 January 2025. Recruitment to the ORT group continued uninterrupted throughout the COVID-19 pandemic. During the inclusion period, parts of the 2011 Norwegian national guidelines were revised in 2019, leading to updated recommendations concerning medication dosage and the selection of pharmacological agents [28]. The group of healthy, unexposed infants were recruited from the maternity ward between March 2018 and March 2019. This study adhered to the STROBE (Strengthening the Reporting of Observational Studies in Epidemiology) checklist to ensure the quality and transparency of the research methodology and reporting.

### Study population

#### ORT group

**Fig. 1:**
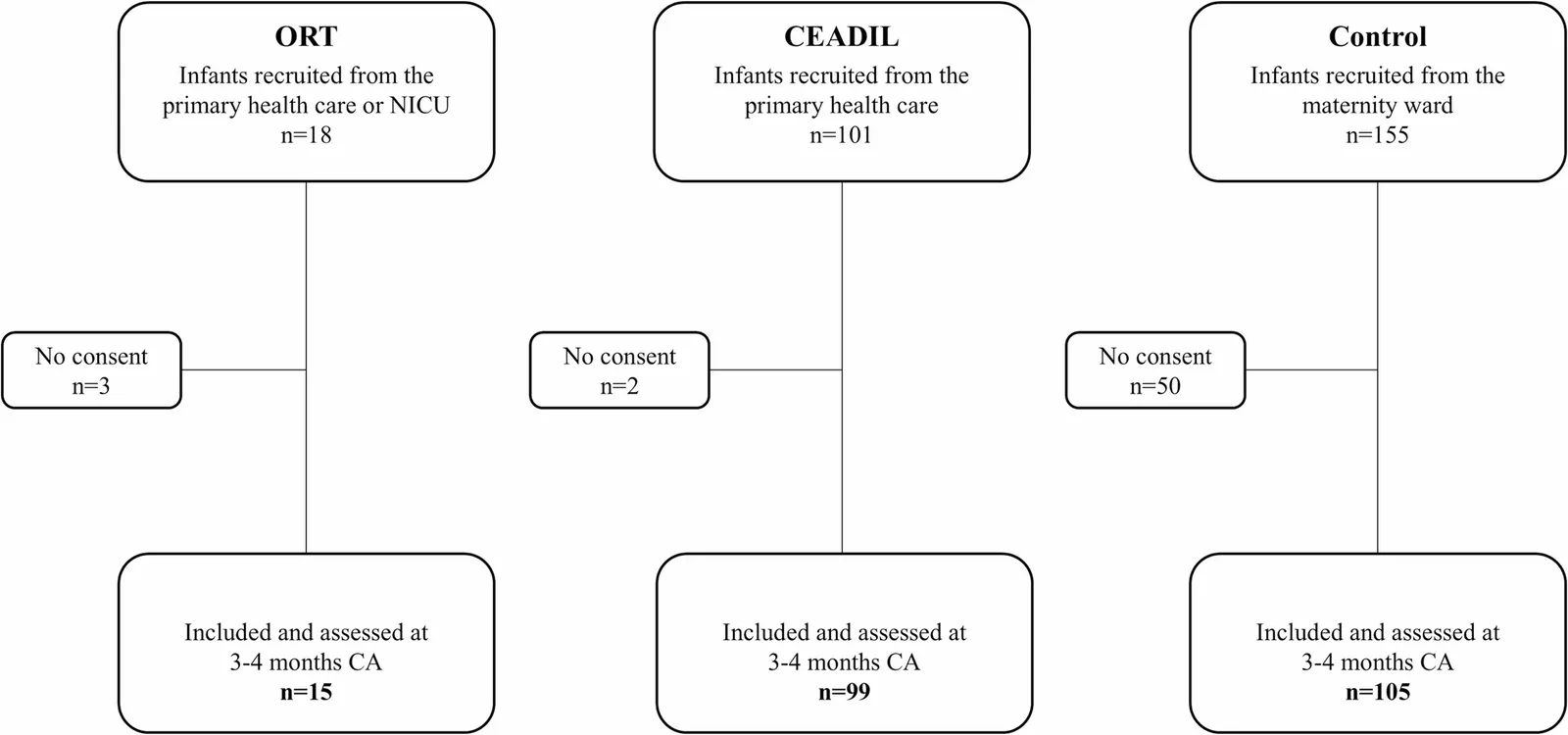
Flow chart of inclusion in the three groups. ORT: Opioid Replacement Therapy. CEADIL: Children Exposed to Alcohol and/or Drugs in Intrauterine Life

All infants in the ORT group were referred to the hospital-follow-up program from the local primary health care (Trondheim, *n* = 12) or the neonatal intensive care unit (NICU) (Oslo, *n* = 3). The mothers had participated in the Norwegian follow up program for pregnant women and had been identified as in ORT during pregnancy by the social services. Social services have a follow up program for all individuals with known substance use disorder. Inclusion criterion in the ORT group was exposure to ORT throughout the entire pregnancy due to maternal treatment. Parents of 15 infants gave their written informed consent to participate on behalf of their child (Fig. 1).

#### CEADIL group

The infants in the CEADIL group were referred to the same hospital-follow-up program from the local primary health care. The mothers were identified by the primary health care or social services as individuals with known substance use disorder or use of prescription drugs for reasons of medical treatment during pregnancy. The inclusion criterion in the CEADIL group was referral to the follow-up program caused by exposure of alcohol and/or drugs during pregnancy. Parents of 99 infants gave their written informed consent to participate on behalf of their child. Information regarding maternal abuse during pregnancy was self-reported by the mothers [14].

#### Control group

The control group was recruited from the maternity ward, before discharge, at St. Olavs hospital, Trondheim University Hospital, Norway between February and December 2018. Parents of 155 infants were invited to participate and 105 gave their written consent on behalf of their child to participate in the control group. The inclusion criteria were healthy, unexposed infants, with normal birth weight, uncomplicated delivery and neonatal period. All included infants in the control group were singletons.

### Clinical characteristics at birth

Clinical characteristic variables such as sex, gestational age, birth weight and length, head circumference at birth, and neonatal complications was obtained from hospital records and/or parents in the ORT and the CEADIL group, and from the parents in the control group.

### Clinical assessment and outcome measures at 3–4 months corrected age

#### Motor Optimality Score – Revised

The MOS-R is a supplementary clinical tool to the GMA, which can be utilized to analyse the infant’s movement, posture and age adequacy [22, 29]. The observers used a standardized scoring form to assess the quality of the infant’s movements. The assessment consists of five subcategories: (i) temporal organization of fidgety movements, (ii) observed movement pattern, (iii) age adequate movement repertoire, (iv) observed postural pattern and (v) movement character [22]. The total score for the MOS-R range from 5 to 28, and score from 25 to 28 is considered as optimal [22].

The infants in our study were video recorded for 3–10 min during the fidgety age, at 9–17 weeks CA. During the recording the infants were active wakeful, lying in a supine position, without interaction. The videos were assessed using the MOS-R-tool by GMA-certified, experienced paediatric physiotherapists in compliance with the procedure described by Einspieler et al. [22]. The observers were not aware of the children’s group allocation. The videos of all three groups were mixed and assessed along with videos of other infants recorded for routine clinical purposes. The observers evaluated the video recordings separately. If there was a disagreement in scoring between the two first observers, the project coordinator assigned the videos to a third observer.

MOS-R total score ≤ 24 was considered to be non-optimal [14, 20, 22, 30]. Absent, sporadic and abnormal fidgety movements were categorized as aberrant fidgety movements and monotonous, stiff and jerky movement character as abnormal movement character.

#### The Alberta Infant Motor Scale

The Alberta Infant Motor Scale (AIMS) is a standardized tool to assess development of postural control in various positions (prone, supine, sitting and standing) in infants from term to 18 months of age [31]. The test is designed to identify infants with motor delay and to evaluate motor performance over time or before/after an intervention [31, 32]. Experienced paediatric physiotherapists conducted the AIMS at the visit concurrent with the GMA recording. The clinicians were not blinded to group allocation during the AIMS assessments, as the children in the ORT and CEADIL groups were evaluated during routine follow-up visits. However, they were not aware of the infants’ scoring/results of the GMA. A chart shows the child’s percentile in relation to a normative, age-matched control sample. AIMS total score below 10th percentile at the age of four months was considered to be atypical motor development [31].

### Statistical analyses

A power calculation was conducted prior to this study, based on the ORT exposed children already included in the CEADIL study (*n* = 13). The calculation was based on findings from Fjørtoft et al. [14], which reported a significant 2-point difference in the MOS-R between the CEADIL and the control group using the Mann–Whitney U test. Given that the scores in the ORT group would be similar as in the CEADIL group, the power calculation indicated that a study sample of 13 participants was sufficient to detect differences between the three groups. We chose to include a few additional participants to increase the statistical power of the study (*n* = 15).

Data were analysed with SPSS Statistics, version 29.0 (IBM SPSS Statistics, Chicago, IL, USA). Normality of residuals were evaluated by visual inspection of Q-Q plots [33]. Despite the normal distribution of certain variables, non-parametric tests were considered the most suitable analytical approach. Continuous data were analysed using the Kruskal-Wallis test for three-group comparisons, and the Mann-Whitney U test to identify which group differed from the other(s) [34]. Differences between the groups in categorical data were analysed using Chi-square test. Odds ratio of 95% confidence interval (CI) was calculated as an estimate of the relative risk of having a reduced quality of early motor behaviour.

### Ethics

The study was conducted in accordance with the Declaration of Helsinki and approved by the Regional Committee for Medical and Health Research Ethics Mid in Norway (REC 2017/2369). All parents or foster parents provided written informed consent after receiving detailed information about the research project. All assessments were observational and non-intrusive to the infants.

## Results

### Clinical characteristics

Clinical characteristics at birth and at assessment at 3–4 months CA are presented in Table 1.

**Table 1:** Clinical characteristics at birth and at assessment

	ORT			CEADIL			Control		
	*n*	Mean	(SD)	*n*	Mean	(SD)	*n*	Mean	(SD)
Gestational age (weeks)	15	38.3	(2.9)	96	38.8	(1.9)	103	39.7	(0.8)
Head circumference at birth (cm)	14	34.2	(1.8)	90	34.6	(1.8)	104	35.5	(1.6)
Birth weight (g)	15	3252	(705)	96	3259	(643)	104	3579	(444)
Birth length (cm)	13	48.8	(2.7)	90	48.4	(2.6)	105	50.2	(1.9)
PMA at assessment (weeks)	15	52.6	(1.8)	99	53.0	(1.3)	105	53.6	(1.3)
Boys, n (%)	15	9	60.0	99	55	55.6	105	46	43.8
Preterm (GA 32 > 37 weeks), n (%)	15	3	20.0	99	10	10.4	105	1*	1.0
Neonatal complications, n (%)	12	0	0.0	99	0	0.0	105	0	0.0
NICU, n (%)	12	3	25.0	99	2	2.0	105	0	0.0
Foster parents, n (%)	15	2	20.0	99	15	15.2	105	0	0.0
Reported “No use”, n (%)	15	0	0.0	99	3	3.0	105	0	0.0
Use of prescription drugs, n (%)	15	0	0.0	99	31	31.3	105	0	0.0
Abuse of alcohol, benzodiazepines, cannabis or amphetamine, n (%)	15	0	0.0	99	65	65.7	105	0	0.0

*ORT* Opioid Replacement Therapy, *CEADIL* Children Exposed to Alcohol and/or Drugs in Intrauterine Life, *PMA* Post Menstrual Age, *NICU* Neonatal Intensive Care Unit

* Late preterm (> 36 weeks)

### Motor Optimality Score–Revised

Table 2 shows the total MOS-R score in the three groups. Both the ORT and the CEADIL groups had a lower MOS-R score compared with the control group (*p* < 0.001). However, there were no significant differences between the ORT group and the CEADIL group.

**Table 2:** Motor Optimality Score–Revised in three groups

	ORT			CEADIL			Control			*p*-value
	*n*	Median	IQR	*n*	Median	IQR	*n*	Median	IQR	
Total score	15	26	(22-28)	98	26	(24-28)	104	28	(26-28)	< 0.001
ORT vs. CEADIL										0.948
ORT vs. Control										0.005
CEADIL vs. Control										< 0.001

*ORT* Opioid Replacement Therapy, *CEADIL* Children Exposed to Alcohol and/or Drugs in Intrauterine Life, *MOS-R* Motor Optimality Score Revised

Motor Optimality List, the list of the subcategories of MOS-R, at 3–4 months CA for all three groups is presented in Table 3, including three-group and two-group comparisons.

**Table 3:** Motor Optimality List in three groups

		ORT *n* = 15		CEADIL *n* = 98		Control *n* = 104		*p*-value
		*n*	%	*n*	%	*n*	%	
i. Fidgety Movement	Normal	12	80.0	87	88.9	100	96.2	0.019
	Abnormal	1	6.7	5	6.1	0	0.0	
	Absent^a^ or sporadic	2	13.3	6	5.1	4	3.8	
ORT vs. CEADIL								0.420
ORT vs. Control								0.021
CEADIL vs. Control								0.024
ii. Observed Movement Patterns	N > A	14	93.3	97	99.0	104	100	0.067
	N = A	1	6.7	0	0.0	0	0.0	
	N < A	0	0.0	1	1.0	0	0.0	
ORT vs. CEADIL								0.249
ORT vs. Control								0.126
CEADIL vs. Control								0.485
iii. Age-Adequate Movement Repertoire	Present	9	60.0	53	54.1	86	82.7	< 0.001
	Reduced	3	20.0	45	45.9	18	17.3	
	Absent	3	20.0	0	0.0	0	0.0	
ORT vs. CEADIL								< 0.001
ORT vs. Control								0.001
CEADIL vs. Control								< 0.001
iv. Observed Postural Patterns	N > A	13	86.7	83	84.7	101	97.1	0.009
	N = A	0	0.0	5	5.1	2	1.9	
	N < A	2	13.3	10	10.2	1	1.0	
ORT vs. CEADIL								0.841
ORT vs. Control								0.056
CEADIL vs. Control								0.003
v. Movement Character	Smooth and fluent	6	40.0	35	35.7	81	77.9	< 0.001
	Abnormal but not cramped-synchronized	9	60.0	63	64.3	23	22.1	
	Cramped-synchronized	0	0.0	0	0.0	0	0.0	
ORT vs. CEADIL								0.778
ORT vs. Control								0.004
CEADIL vs. Control								< 0.001

^a^No infants had absent fidgety movements

*ORT* Opioid Replacement Therapy, *CEADIL* Children Exposed to Alcohol and/or Drugs in Intrauterine Life

In all but one subcategory (observed movement pattern) of MOS-R, a higher proportion in both the ORT and the CEADIL groups had poorer results compared with the control group (Table 3). Three (20.0%) infants in the ORT group had abnormal or sporadic fidgety movements, compared with 11 (11.2%) in the CEADIL group and four (3.8%) in the control group. None of the infants in the three groups had absent fidgety movements. There were no differences between the three groups in observed movement patterns. Three (20.0%) infants in the ORT group had absent age-adequate movement repertoire, compared with none in the CEADIL group and control group. Two (13.3%) in the ORT group and 10 (10.2%) in the CEADIL group had more abnormal than normal observed postural patterns (N < A), compared with one (1.0%) in the control group. Nine (60.0%) infants in ORT group had abnormal movement character, compared with 63 (64.3%) in CEADIL group and 23 (22.1%) in the control group.

### Alberta Infant Motor Scale

Infants in the ORT group had a median total AIMS score of 8.0 (IQR 7.0-11.5), compared with 9.0 (IQR 8.0–10.0) in the CEADIL group and 11.8 (IQR 11.0–13.0) in the control group. There was no significant difference between the ORT group and the CEADIL group, but both groups had a lower median total AIMS score compared with the control group (*p* < 0.001, Table 4).

**Table 4:** Total score of Alberta Infant Motor Scale in three groups

	ORT			CEADIL			Control			
	*n*	Median	(IQR)	*n*	Median	(IQR)	*n*	Median	(SD)	*p*-value
Total score	15	8.0	(7.0-11.5)	96	9.0	(8.0-10.0)	99	11.8	(11.0-13.0)	< 0.001
ORT vs. CEADIL										0.97
ORT vs. Control										< 0.001
CEADIL vs. Control										< 0.001

*ORT* Opioid Replacement Therapy, *CEADIL* Children Exposed to Alcohol and/or Drugs in Intrauterine Life, *AIMS* Alberta Infant Motor Scale

### Odds of reduced quality of early motor behaviour

Table 5 presents the odds ratio (OR) for reduced motor behaviour in the three groups. More infants in both the ORT and the CEADIL groups had a non-optimal MOS-*R* ≤ 24 and substantially higher odds for scoring below cut-off, adjusted for GA and sex, compared with the unexposed control group (Table 5). The exposed groups also had higher odds for abnormal movement character and aberrant fidgety movements, compared with the control group.

**Table 5:** Odds ratio for reduced quality of early motor behaviour in three groups

			Crude		Adjusted for GA		Adjusted for sex		Adjusted for GA and sex	
	*n*	(%)	OR	(95% CI)	OR	(95% CI)	OR	(95% CI)	OR	(95% CI)
MOS-*R* ≤ 24										
ORT	6	40.0	5.6	(1.7-18.9)	4.6	(1.3-16.1)	5.5	(1.6-18.4)	4.3	(1.2-15.2)
CEADIL	43	43.9	6.4	(3.0-13.4)	5.5	(2.6-11.9)	6.4	(3.0-13.4)	5.3	(2.4-11.5)
Control	11	10.6	1		1		1		1	
Aberrant Fidgety Movements										
ORT	3	20.0	6.3	(1.2-31.3)	6.4	(1.2-338)	5.8	(1.1-29.3)	5.5	(1.0-29.4)
CEADIL	11	11.2	3.1	(1.0-10.2)	3.2	(1.0-10.9)	2.9	(0.9-9.6)	2.9	(0.9-10.0)
Control	4	3.8	1		1		1		1	
Abnormal Movement Character										
ORT	9	60.0	5.3	(1.7-16.4)	4.6	(1.4-14.9)	5.1	(1.6-16.0)	4.3	(1.3-14.0)
CEADIL	63	64.3	6.2	(3.3-11.4)	5.7	(3.0-11.0)	6.0	(3.2-11.2)	5.5	(2.9-10.5)
Control	23	22.1	1		1		1		1	
AIMS total score < 10th percentile										
ORT	4	26.7	16.7	(1.7-169.1)	14.2	(1.4-143.4)	17.0	(1.7-169.2)	14.1	(1.4-145.2)
CEADIL	21	21.9	14.7	(1.9-112.6)	12.8	(1.7-99.8)	14.9	(1.9-114.7)	12.8	(1.6-100.5)
Control	0	0.0	1		1		1		1	

*MOS-R* Motor Optimality Score-Revised, *ORT* Opioid Replacement Therapy, *CEADIL* Children Exposed to Alcohol and/or Drugs in Intrauterine Life, *AIMS* Alberta Infant Motor Scale

More than 20% of the infants in the ORT and the CEADIL group had a total score on the AIMS < 10th percentile, compared with none in the healthy, unexposed control group (*p* < 0.001). This gives an extensively higher odds for atypical motor development with OR of 14.1 (95% CI: 1.4-145.2) in the ORT group and OR of 12.8 (95% CI: 1.6-100.5) in the CEADIL group, adjusted for GA and sex.

## Discussion

### Main findings

To our knowledge, the present study is the first to investigate early motor behaviour in ORT- exposed infants at 3–4 months CA using MOS-R and the AIMS. We found that infants born to mothers in ORT during pregnancy exhibited reduced quality of early motor behaviour, similar to infants exposed to alcohol and/or drugs during pregnancy, compared with a group of healthy, unexposed infants. Infants in both the ORT and the CEADIL groups had lower MOS-R scores, a higher frequency of aberrant fidgety movements, reduced or absent age-adequate movement repertoire, abnormal observed postural patterns, and abnormal movement character, compared with the unexposed control group. Both the ORT and the CEADIL groups had lower scores on the AIMS. In both groups, the odds of reduced quality of motor behaviour were substantially higher when assessed with the MOS-R and the AIMS.

### Strengths and limitations

Strengths of this study are the prospective design and inclusion of both ORT and CEADIL groups, along with an unexposed group of infants, which gives the opportunity to follow these infants during the development from the age of 3–4 months and hopefully until school age. This can give valuable information about the child’s neurodevelopment, including long-term consequences. The prospective design can aid in identifying early markers for at-risk children and in detecting the predictive values of assessment tools.

Few ORT-exposed infants are born annually, due to the recommendation for women in ORT to avoid pregnancy. Hence, the number of participants in the ORT group was limited (*n* = 15). Still, the included children may be regarded as representative for ORT-exposed children because of the comprehensive follow-up program for these mothers. Thus, it can be assumed that the vast majority of eligible infants in the catchment area of St. Olavs hospital are included. Children from Oslo University Hospital, Ullevål, were included to increase the number of participants, as they have a larger catchment area. Still, non-significant findings should be interpreted with caution due to the size of the ORT group.

Another key strength of our study is that the assessments of the infants were conducted by experienced clinicians, certified GMA observers and the GMA assessments were performed in a blinded manner. The inter- and intra-rater reliability for the AIMS and the MOS-R is both considered to be excellent [23, 35, 36]. Furthermore, the assessment tools used were well-known and validated instruments, both designed to detect neurological and atypical motor development, which contribute to the study’s strength. Spittle et al. [35] concludes in their systematic review that the GMA have the greatest combination of sensitivity and specificity in the first months of life, and the AIMS in the later months. Unfortunately, there are no normative data available for the Norwegian infants aged 3–4 months, but a recently validation study of Norwegian infants at 6–9 months CA concluded that either Canadian or Dutch normative data are valid for Norwegian infants [37]. Hence, there is still a need to establish normative values for Norwegian children aged 0–18 months CA.

However, the study has certain limitations. We lack information on which specific medical drugs were used (methadone or buprenorphine), the dosage administered in ORT, and the adherence of mothers to ORT during pregnancy. Although all the mothers in the ORT group were in ORT during the whole pregnancy, resulting in relatively stable opioid levels and potentially less stress for the foetus, the possibility of dose reduction during pregnancy cannot be ruled out. National guidelines [1] advise against tapering the medical dosage during pregnancy, yet there is no consensus among medical professionals regarding the benefits or risk reduction for both mother and foetus [38]. Moreover, there is still no conclusive research on the ideal dosage for both mother and foetus during pregnancy nor which medical drug is recommended [39]. A recently published Norwegian study [17] found a tendency towards better outcomes for children with lower drug exposure during ORT in all domains tested, at the age of five to 13 years. Another study found significant poorer motor outcomes with higher methadone dose during pregnancy at the age of two years [40] and a third reported a linear relationship between the methadone dose and natal and infant clinical outcome [41]. In light of this, further research on the dosage of substitution treatment during pregnancy and the child’s short- and long-term outcomes is needed, with the aim of establishing clearer consensus in the treatment and follow-up of pregnant mothers and their unborn children.

Another limitation is the reliance on self-reported data for drug use and ORT in both the ORT and CEADIL groups. An American study [42] has shown substantial underreporting for most major drug classes among pregnant women, although reporting is more accurate among those in ORT or using marijuana. Hence, it is reasonable to assume that there is an underreporting of drug use or additional substance use by the mothers in both the ORT group and the CEADIL group. This factor could potentially affect the accuracy of the findings related to drug exposure.

We must take into account that there may be confounding factors affecting our results. We have no information on maternal smoking, maternal, perinatal, and postnatal stress, maternal food intake, or detrimental postnatal care environment, except that some of the children in the ORT and the CEADIL group live in foster homes. All of these factors may influence foetal and infant growth and development [43–47].

### Early motor behaviour

None of the infants exhibited absence of fidgety movements by the GMA, which has been shown to be a predictive factor for the development of cerebral palsy [48–52]. Therefore, it can be argued that there is a low risk for the development of cerebral palsy among the children included in this study. However, several of the children exhibited aberrant fidgety movements, which is a less predictive finding, but still suboptimal. In the CEADIL follow-up study [15] we found that presence of suboptimal fidgety movements identified almost all exposed children with NDI at the age of two years, but with low specificity. The utility of the combination between FMs, movement character and MOS-R were highlighted. Some studies have nevertheless shown that children with abnormal fidgety movements are at risk of minor NDI such as autism spectrum disorders [27, 53] or developmental delay and attention deficit hyperactivity disorder at age 2–3 years [27]. This provides us with reason to suggest that children with aberrant fidgety movements should be monitored further through their development.

There were no differences between the ORT and the CEADIL group on GMA and AIMS, except for the MOS-R subcategory age-adequate movement repertoire, where the ORT group had lower scores compared with the CEADIL group. This may indicate that the subcategory age-adequate movement repertoire of the MOS-R, may be more sensitive in detecting differences in early motor behaviour in this age group than the other MOS-R subcategories and the AIMS. In the subcategory age-adequate movement repertoire in the MOS-R, children are assessed based on 23 movement patterns [22], compared to relatively few items in the AIMS [31]. This is also supported by Yilidrim’s et al. [54] conclusion that the AIMS is more sensitive between six and 12 months. In our present study both the ORT and the CEADIL groups had a substantially higher risk for abnormal movement character, adjusted for GA and sex, compared with the unexposed infants. [55] reported that Bos et al. found  that a monotonous character of motor behaviour increased the probability of the development of mild NDI, such as tonus abnormalities, coordination problems and gross motor problems. This subcategory can therefore help identify children who need closer follow-up.

Our findings showed that both the exposed groups of infants had a significantly lower total score of MOS-R and higher odds of a suboptimal MOS-R with total scores ≤ 24, compared with the unexposed infants. Kwong et al. [24] found a strong relationship between the MOS-R score and NDI at two years in children born extremely preterm and/or with extremely low birth weight, where a higher score was related to reduced odds of NDI. They suggest that the MOS-R has a clinical value in identifying infants at risk for motor, cognitive and/or language developmental impairment. They also refer to the MOS-R as a more nuanced measure of early motor repertoire, particularly more sensitive for identifying children with non-CP developmental delays, compared to the GMA alone. This can indicate that more of the infants, in both the ORT and the CEADIL groups, are at risk for NDI later in life. Still, due to the research of Fjørtoft et al. [15] the MOS-R with a cut-off score ≤ 24 has limited sensitivity identifying only half of the exposed children with NDI at two years follow up. It may therefore be valuable to consider which cut-off value on the MOS-R is most appropriate for identifying children at risk for NDI, while avoiding unnecessary concern for parents of children with typical development. Additionally, it is also valuable to reassure the parents of children who achieve a high optimality score on the MOS-R.

### Biological credibility

It is possible that reduced quality of motor behaviour in the ORT group is an expression of delayed maturation in the nervous system due to residual symptoms from NAS, which can persist until 4–6 months of age [8]. Unfortunately, there was no consistent reporting on how many of the children were diagnosed with NAS after birth in this study. Furthermore, only few children were treated in the NICU due to withdrawal symptoms, and no other peri- or neonatal complications were reported.

In light of the understanding of critical brain development processes during pregnancy and the first three months after birth [55, 56], and with general movements recognized as an excellent marker for early brain impairment and dysfunction [51], the findings in the present study may indicate alterations in the infants’ brain development due to exposure of ORT, alcohol and/or drugs during maternal treatment. These findings align with previous research demonstrating that prenatal exposure to ORT or alcohol is associated with alterations in newborn brain structure and brain volume [57, 58]. Furthermore, these studies have shown that such structural alterations are correlated with later neurodevelopmental outcomes [57, 58]. Taken together, this body of evidence supports the interpretation that the reduced early motor behaviour in both the ORT and CEADIL groups may reasonably be attributed to the exposure during pregnancy.

### Clinical implications

This study provides valuable insights into the impact of ORT, alcohol, and drug exposure in pregnancy on early motor behaviour. The findings of group differences in quality of early motor behaviour highlight that exposed infants are at risk for NDI and should be monitored by healthcare professionals familiar with these challenges. Utilizing the MOS-R and the AIMS as motor assessment tools in this population can aid in identifying infants at risk of adverse development, facilitating the provision of targeted healthcare services from an early age, which is cost-effective for society. Furthermore, the MOS-R can be used to identify infants with typical development, offering reassurance to their parents.

Despite findings of reduced quality of early motor behaviour in both the ORT and CEADIL groups, we cannot definitively determine whether this reduction in motor behaviour is permanent or temporary. Further research should investigate the long-term effects of exposure to ORT, alcohol and/or drugs during pregnancy and the relationship with early motor behaviour and later development.

## Conclusions

Similar to the CEADIL group, the ORT group had reduced quality of early motor behaviour and had substantially higher odds for reduced motor behaviour compared with the control group at 3–4 months CA. Hence, ORT-exposed infants may be at risk for neurodevelopmental impairment.

## Data Availability

The datasets used and/or analysed during the current study are available from the corresponding author on reasonable request. The GMA videos are not available for ethical and regulatory reasons.

## Abbreviations

ORT: Opioid Replacement Therapy
.: Opioid Maintenance Therapy
.: Methadone Maintenance Treatment
MOS-R: Motor Optimality Score-Revised
AIMS: Alberta Infant Motor Scale
NAS: Neonatal Abstinence Syndrome
.: Neonatal Opioid Withdrawal Syndrome
NDI: Neurodevelopment Impairment
.: Neurological Impairment
.: Neurodevelopmental Delay
GMA: General Movement Assessment
FM: Fidgety Movement
CP: Cerebral Palsy
PMA: Post Menstrual Age
CA: Corrected Age
OR: Odds Ratio
